# Oxytocin increases eye contact during a real-time, naturalistic social interaction in males with and without autism

**DOI:** 10.1038/tp.2014.146

**Published:** 2015-02-10

**Authors:** B Auyeung, M V Lombardo, M Heinrichs, B Chakrabarti, A Sule, J B Deakin, R A I Bethlehem, L Dickens, N Mooney, J A N Sipple, P Thiemann, S Baron-Cohen

**Affiliations:** 1 Department of Psychology, The School of Philosophy, Psychology and Language Sciences, University of Edinburgh, Edinburgh, UK; 2 Autism Research Centre, Department of Psychiatry, University of Cambridge, Cambridge, UK; 3 Department of Psychology, University of Cyprus, Nicosia, Cyprus; 4 Center for Applied Neuroscience, University of Cyprus, Nicosia, Cyprus; 5 Department of Psychology, Laboratory for Biological and Personality Psychology, University of Freiburg, Freiburg, Germany; 6 Freiburg Brain Imaging Center, University Medical Center, University of Freiburg, Freiburg, Germany; 7 School of Psychology and Clinical Language Sciences, University of Reading, Whiteknights, Reading, UK; 8 South Essex Partnership University NHS Foundation Trust, Bedford, UK; 9 Department of Psychiatry, University of Cambridge, Cambridge, UK; 10 Cambridgeshire and Peterborough NHS Foundation Trust (CPFT), Cambridge, UK

## Abstract

Autism spectrum conditions (autism) affect ~1% of the population and are characterized by deficits in social communication. Oxytocin has been widely reported to affect social-communicative function and its neural underpinnings. Here we report the first evidence that intranasal oxytocin administration improves a core problem that individuals with autism have in using eye contact appropriately in real-world social settings. A randomized double-blind, placebo-controlled, within-subjects design is used to examine how intranasal administration of 24 IU of oxytocin affects gaze behavior for 32 adult males with autism and 34 controls in a real-time interaction with a researcher. This interactive paradigm bypasses many of the limitations encountered with conventional static or computer-based stimuli. Eye movements are recorded using eye tracking, providing an objective measurement of looking patterns. The measure is shown to be sensitive to the reduced eye contact commonly reported in autism, with the autism group spending less time looking to the eye region of the face than controls. Oxytocin administration selectively enhanced gaze to the eyes in both the autism and control groups (transformed mean eye-fixation difference per second=0.082; 95% CI:0.025–0.14, *P*=0.006). Within the autism group, oxytocin has the most effect on fixation duration in individuals with impaired levels of eye contact at baseline (Cohen's *d*=0.86). These findings demonstrate that the potential benefits of oxytocin in autism extend to a real-time interaction, providing evidence of a therapeutic effect in a key aspect of social communication.

## Introduction

Autism spectrum conditions (henceforth autism) are characterized by deficits in social communication alongside unusually narrow interests and repetitive behaviors. There are currently few effective behavioral or pharmacological treatments that can ameliorate core social difficulties associated with autism. Little is also known about how treatments may be effective for subgroups within autism; so far, none have been developed with specificity in mind for targeting a specific subset of behaviors that individuals on the autism spectrum may have particular difficulty with. One exemplar domain of social behavior that would be a critical target for treatments is eye gaze behavior and processing gaze information of others. Eye gaze is observed to be atypical in many cases of autism from early infancy and persists into adulthood.^1, 2, 3, 4, 5^ Developmentally, sensitivity to information from the eyes and appropriate use of gaze behavior in social contexts may also provide an important platform for later development of more complex social-cognitive abilities that are requisite for further development of social cognition and behavior.^6, 7^ Given the importance of gaze processing and behavior, treatments that can target this aspect of the phenotype may offer much help to individuals with autism.

One candidate pharmacological target of much current interest for autism and social behavior is the neuropeptide oxytocin. A large body of research across human and nonhuman species has demonstrated a role for oxytocin in social behavior and cognition.^8, 9, 10, 11, 12, 13, 14, 15, 16^ One example of this is seen in oxytocin's influence on eye gaze behavior. Oxytocin administration has been shown to increase gaze toward the eye region of static photographs of the face,^14^ and also increases orienting of attention to gaze cues.^17^ fMRI studies have also found that oxytocin exerts influence on important neural circuits for a wide range of social-cognitive abilities such as gaze, mentalizing, emotion-recognition and learning.^11, 18, 19, 20, 21, 22, 23, 24, 25^ Oxytocin has also been evaluated in studies of autism, and has been shown to have a broad impact on an array of important behaviors and neural systems.^10, 26, 27, 28, 29, 30^ Abnormalities in genetic variation of the oxytocin receptor,^13, 31^ as well as animal models^32^ have also provided evidence that oxytocinergic abnormalities exist in autism.

However, despite the mounting evidence for the importance of oxytocin as a potential pharmacological target for further development in terms of how it might affect aspects of social behavior in autism, all of the work until now has been based on studies done in constrained laboratory settings. These studies are obviously necessary and critical. However, for furthering the translational process, there is also a need to test whether the effects of oxytocin can have substantial impact on social-communicative behavior in more naturalistic real-world social contexts. In addition, translational progress must be made for examining how oxytocin may or may not be effective for certain individuals and to better characterize who might benefit the most from oxytocin.

The present study aims to address these issues by evaluating the effect of oxytocin on gaze behavior during a naturalistic live, real-life social interaction with another person. Furthermore, we examined how oxytocin might be more or less effective in subgroups of individuals with autism who exhibited either below average eye contact (relative to the control group), intact, or above average eye contact under baseline placebo conditions.

## Materials and methods

A randomized, double-blind, placebo-controlled, within-subjects design was used. Independent pharmacists dispensed either an active or placebo spray according to a computer-generated randomization list. Participants received an active intranasal oxytocin spray (24 IU, 40.32 μg, Syntocinon-spray; Novartis, Switzerland), three actuations administered to each nostril, (4I U, 6.72 μg each) or a placebo containing the same ingredients with the exception of the active oxytocin, on two visits separated by 1 week. The single 24 IU dose was selected on the basis of findings from earlier studies of oxytocin and behavior suggesting that a similar dose of oxytocin could enhance social behavior compared with placebo (for reviews, see Heinrichs *et al.*^13^ and Meyer-Lindenberg *et al.*^33^).

### Participants

Males 18–56 years of age (*M*=34.23, s.d=9.18) were included in this study. The clinical group consisted of 37 males with a diagnosis of Autism or Asperger Syndrome based on strict DSM-IV criteria.^34^ Thirty-seven typically developing males were recruited from the University of Cambridge and the general population. Exclusion criteria included smoking; a diagnosis of major depression, bipolar, obsessive–compulsive, panic or psychotic disorder; use of any psychoactive medication within one year of the study; substance dependence; or epilepsy. Women were excluded to avoid sex differences in oxytocin response.^9, 12^

### Measure

The study evaluated naturalistic social behavior during a semi-structured interview with a researcher at a remote location, facilitated by video link. This allowed the use of eye tracking with a Tobii T120 binocular infrared eye tracker, which records the x and y coordinates of the participant's eye position using corneal reflection techniques. The camera is embedded in a 17-inch thin-film transistor LCD monitor (1280 × 1024 pixels resolution), promoting more naturalistic participant behavior since it does not place restraints on participants such as a head mount or glasses. The eye tracker samples the position of the eyes at a rate of 120 Hz, with an average precision of within 0.5-degree visual angle.^35^

At both the visits, participants were asked questions about their well-being, their journey to the research center, how the nasal spray made them feel, attitudes towards participating in research and what they liked and did not like about the study.

At the interviewer's location, the camera was positioned directly ahead of the screen so as to be level with the image of the participant's eye level. The interviewer was trained to look directly into the camera to ensure that the participant perceived the interviewer was providing eye contact. The same researcher performed the interviews for both visits. Considerable attention was given to ensuring consistency in the visual appearance of the interviewer between visits, and a plain white background was used to minimize the potential for visual distraction.

**Figure 1:**
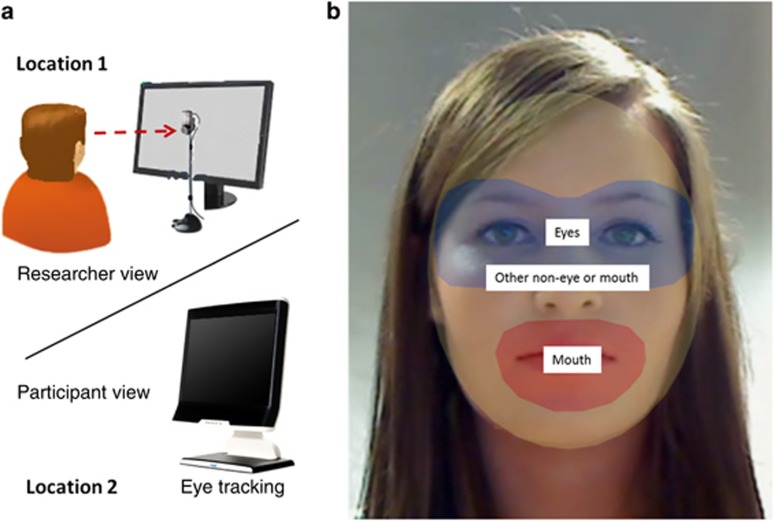
Experimental setup and areas of interest (AOI). (**a**) Depicts the study setup. (**b**) Shows the AOIs drawn for the eyes, mouth and other non-eye-or-mouth regions.

Following completion of the interview, moving areas of interest (AOIs) were drawn around the eyes, mouth, or other non-eye-or-mouth face areas using the ‘Dynamic AOI (area of interest)' tool, using freeform shapes in Tobii Studio.^35^ The dynamic AOIs move with the interviewer's face in real time. See Figures 1a for a representation of the study setup. Figure 1b shows examples of the AOIs drawn for the eyes, mouth and other non-eye-or-mouth regions. Measures included fixation count (number of gaze fixations toward an AOI) and gaze duration (total milliseconds spent fixating on an AOI).^35^

### Procedure

The participants were instructed to abstain from alcohol and caffeine on the day of testing and from food and drink, except water, for 2 h before spray administration.

Upon arrival, a medical examination including measurements of blood pressure, heart rate and general well-being was conducted. A medical professional provided a prescription and remained in attendance to manage any potential ill effects from the spray. The participants received either the active or placebo nasal spray (three puffs per nostril, alternating between sides) and were asked to wait for 45 min^9^ before being asked to complete a questionnaire and a series of computer-based tasks, lasting ~35 min.

The participants were then asked to speak to a researcher at a remote location about their experience with the study, and the interaction was initiated as a semi-structured interview via video link designed to last ~5 min. At the end of the session, the participants were asked to complete a ‘Wellness' questionnaire that asked about side effects that the participants may have experienced. The same experimenter conducted the interview for both visits.

Written consent was obtained before and during the visit. Ethical approval was provided by the UK National Research Ethics Service. This study was exempt from clinical trials status by the UK Medicines and Healthcare products Regulatory Agency.

### Data analysis

The number of fixations and total fixation duration were examined for the eyes, mouth and other non-eye-or-mouth face areas using Tobii Studio software (version 3.2.1). The Tobii I-VT filter (classifier: 30° per second; Velocity calculator window length: 20 ms) was used to identify fixations. No gap-filling or other noise reduction technique was used. Minimum fixation duration was 60 ms. Maximum time between fixations classified as separate fixations was 75 ms. Data were normalized for length of interview as follows:

Normalized number of fixations (per second)=Number of fixations/Interview time

Normalized fixation time (proportion)=Total fixation time/Interview time

A square-root transformation was employed for all the data as they were positively skewed. The transformed data approximated the normal distribution, and transformed data were used in all subsequent analyses.

A repeated-measures analysis of variance (ANOVA) was conducted for a possible three-way interaction between drug (two levels—oxytocin and placebo), AOI (three levels—eyes, mouth other non-eye-or-mouth face areas) and group (two levels—autism and controls). For significant interactions, separate follow-up repeated-measures ANOVAs for each region were conducted to discern the nature of the interaction. For significant interactions, tests within each variable were used to characterize the nature of the interaction.

Finally, the autism group was subdivided to evaluate whether oxytocin had a greater effect on individuals with lower eye contact (measured by fixations to the eye region of the face). Subgroups with ‘Low' and ‘High' eye contact were defined by comparing measured values with the mean score from the control group under placebo, objectively classifying each individual as having lower or higher looking than the average control participant. Individuals with autism were allocated to the ‘Low' group where they scored below the control group mean with both placebo and oxytocin, and to the ‘High' group where they scored above this value with both placebo and oxytocin. Individuals who had scores from each condition, which fell both above and below the control group mean were not included in this analysis, as these individuals could not be clearly classified as belonging to the ‘Low' or ‘High' group. A ‘difference score' of oxytocin—placebo was then computed for the eye AOI. Independent samples *t*-tests with bootstrapping (10 000 resamples) were used to compare the ‘Low' versus ‘High' subgroups using this difference score.

## Results

Several participants were excluded from the final analysis. In the control group, one participant did not attend the second visit, and there was a technical malfunction for two participants. In the autism group, three participants did not attend the second visit, and there was a technical malfunction for two participants. The final data set consisted of 34 control males and 32 males with autism, matched on age and IQ (intelligence quotient using the Wechsler Abbreviated Scale of Intelligence). See Table 1 for descriptive information.

### Case–control analysis for looking preferences (placebo only)

To look at general group differences, comparisons were made between cases and controls under the placebo condition only. Independent samples *t*-tests were conducted, and the means, standard deviations, range, *t*-test and *P*-values are presented in Supplementary Table 1.

**Figure 2:**
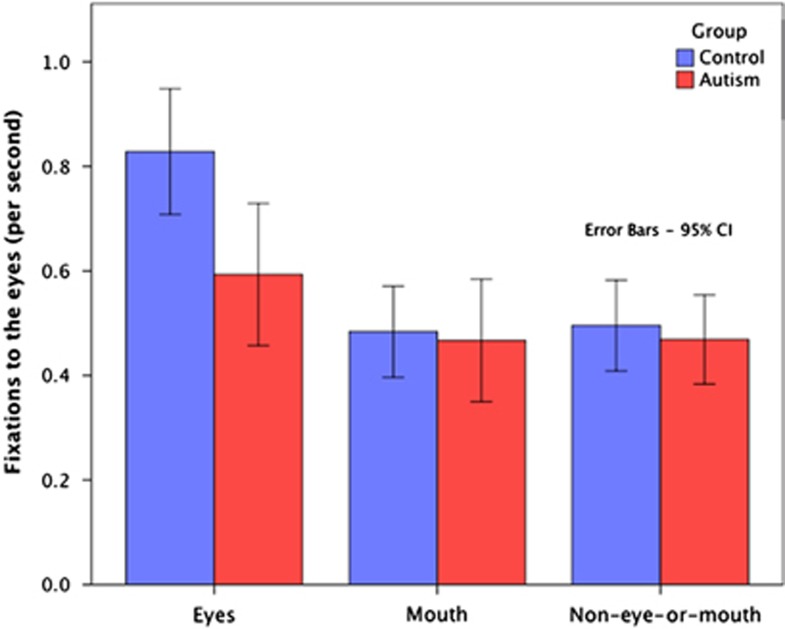
Number of fixations for each AOI by group (normalized for interview length). AOI, area of interest.

Individuals with autism (*M*=0.59, s.d.=0.38) show significantly fewer fixations than controls (*M*=0.83, s.d.=0.34) to the eye region of the face, *t*(64)=2.660, *P*=0.010, Cohen's *d*=0.666. However, no significant group differences were observed for the mouth or non-eye-or-mouth face regions. Figure 2 shows the number of fixations (normalized for interview length) for the eyes, mouth and non-eye-or mouth face areas of interest. Error bars indicate 95% confidence intervals.

There was also a significant difference *t*(64)=2.454, *P*=0.017, Cohen's *d*=0.581 in looking time to the eye region of the face. Individuals with autism showed lower proportion of total looking time to the eyes (*M*=0.32, s.d.=0.23) than controls (*M*=0.44, s.d.=0.18). There were no group differences in looking time to the mouth or the non-eye-or-mouth regions (*P*>0.05).

### Effect of oxytocin—number of fixations

**Figure 3:**
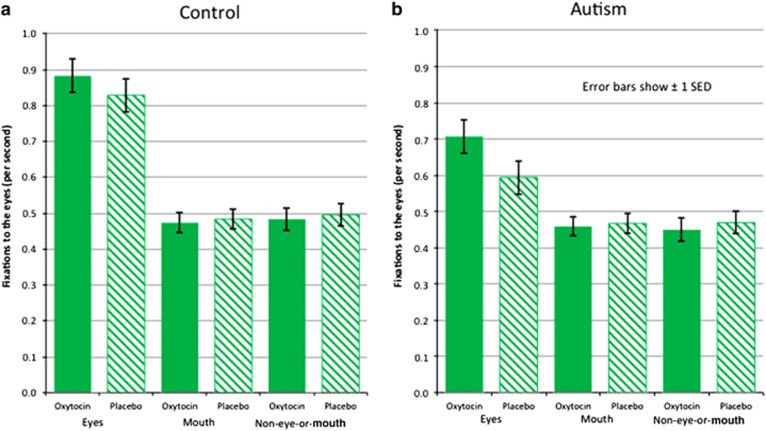
Normalized number of fixations for each AOI by drug (data not transformed). (**a**) Shows fixations to the eyes for each AOI by drug for the control group. (**b**) Shows fixations to the eyes for each AOI by drug for the autism group. Error bars show±1 s.e.d. for this within-subjects comparison. AOI, area of interest.

To understand the relationship between group, drug and looking preferences, a three-way repeated-measures ANOVA was first performed using the transformed data. The three-way ANOVA interaction between drug × AOI × group was not significant (Wilks' Lambda=0.989, F(2,63)=0.352, *P*=0.705, partial *η*^2^=0.011). The two-way interaction between AOI × group was also not significant (Wilks' Lambda=0.952, F(2,63)=1.590, *P*=0.212, partial *η*^2^=0.048). However, the drug × AOI interaction was significant (Wilk's Lambda=0.899, F(2,63)=3.544, *P*=0.035, partial *η*^2^=0.101, Cohen's *d*=0.670). This suggested that there was an effect of drug on specific AOIs. There was also a significant effect for the between-subjects factor (group) (F(1,64)=6.219, *P*=0.015, partial *η*^2^=0.089). Figure 3 shows the number of fixations for each AOI by drug.

Follow-up tests for each AOI show a significant main effect of drug for the eye region (F(1,65)=8.225, *P*=0.006, partial *η*^2^=0.112, Cohen's *d*=0.710), with a larger number of fixations under oxytocin (*M*=0.797, s.d.=0.358) than placebo (*M*=0.7144, s.d.=0.377). No main effects were observed for the mouth region (F(1,65)=0.247, *P*=0.621) or other non-face-or-mouth regions (F(1,65)=0.477, *P*=0.492).

### Total fixation time

For total fixation time, the three-way ANOVA interaction between drug × AOI × group interaction was not significant (Wilks' Lambda=0.999, F(2,63)=0.025, *P*=0.975, partial *η*^2^=0.001). The two-way interaction between AOI × group was also not significant (Wilks' Lambda=0.968, F(2,63)=1.028, *P*=0.364, partial *η*^2^=0.032). The drug × AOI interaction was significant (Wilk's Lambda=0.893, F(2,63)=3.782, *P*=0.028, partial *η*^2^=0.107, Cohen's *d*=0.692), and suggested that there was an effect of drug on specific AOIs. As for the number of fixations, the between-subjects factor (group) was also significant (F(1,64)=10.482, *P*=0.002, partial *η*^2^=0.141).

Follow-up tests for each AOI show a significant main effect of drug for the eye region (F(1,65)=5.607, *P*=0.021, partial *η*^2^=0.079, Cohen's *d*=0.586), with longer looking times under oxytocin (*M*=0.421, s.d.=0.199) compared with placebo (*M*=0.381, s.d.=0.213). No main effects were observed for the mouth region (F(1,65)=1.666, *P*=0.201) or other non-face-or-mouth regions (F(1,65)=0.754, *P*=0.388).

### Within-group analysis in autism

In the autism group, *n*=18 participants had a number of fixations to the eye region which was below the control group placebo mean (Low group) under both oxytocin and placebo, whereas, for *n*=11 participants, both scores were above this value (High group). The effect of oxytocin was evaluated using ‘difference scores', calculated by subtracting the placebo value from the oxytocin value. This analysis indicated no significant difference in oxytocin effect on the number of eye fixations in the ‘Low' group (*M*=0.134, s.d.=0.148) compared with the ‘High' group (*M*=0.044, s.d.=0.180, *t*(27)=1.467, bootstrapped *P*=0.040, Cohen's *d*=0.188).

**Figure 4:**
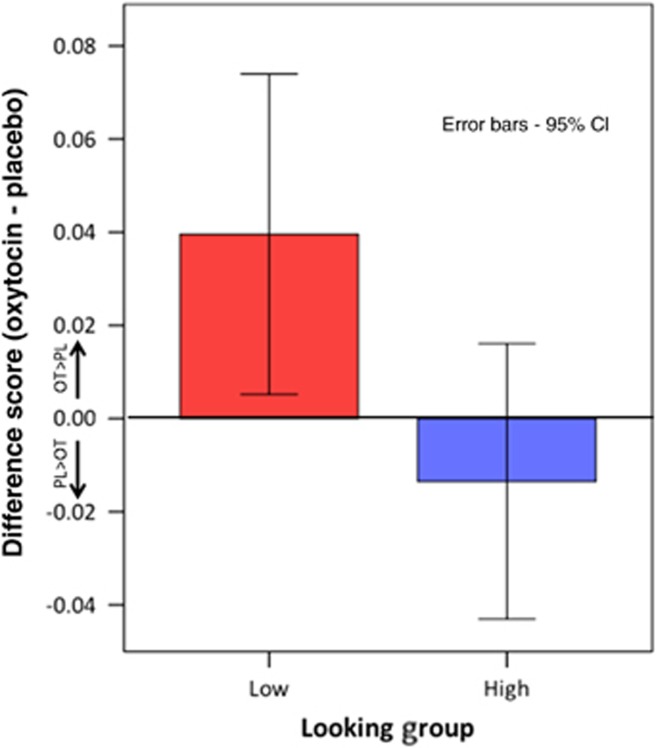
Difference scores in fixation time for the ‘Low' versus ‘High' looking groups in autism (groups identified using control group mean). CI, confidence interval.

For total fixation time, *n*=18 individuals with autism fell into the ‘Low' group, with *n*=9 individuals in the ‘High' group. This showed a greater effect for oxytocin in the ‘Low' group difference score (*M*=0.059, s.d.=0.087) compared with the ‘High' group (*M*=−0.012, s.d.=0.077), *t*(25)=2.083, bootstrapped *P*=0.037, Cohen's *d*=0.864. These data are illustrated in Figure 4.

Individuals who had scores in the oxytocin and placebo condition that fell both above and below the control group mean were not included in this analysis (*n*=3). The difference scores for these participants for the number of fixations and fixations time were *M*=0.241, s.d.=0.376 and *M*=0.146, s.d.=0.248, respectively, and suggest that the exclusion of these participants did not bias the result.

### Side effects

A record was kept of the side effects that the participants experienced from the oxytocin or placebo nasal sprays. No serious adverse effects were reported. Supplementary Table 2 shows the number of participants in each group who reported each side effect.

## Discussion

This double-blind, placebo-controlled study utilized a novel measurement technique to evaluate the effect of oxytocin on gaze behavior in autism (Figure 1). The design of the experiment enabled gaze data to be collected during a real-time social interaction, while also maintaining the repeatability necessary to evaluate drug versus placebo effects. Findings from the baseline (placebo) condition illustrate the reduced eye contact that is frequently observed in autism (Figure 2). These findings are in accordance with work showing reduced eye contact in autism,^36^ and suggest that the measure used is ecologically valid.

Both the oxytocin and placebo nasal sprays were successfully administered to all of the participants, with few side effects reported. Oxytocin was found to significantly increase participant gaze to the eye region of the interviewer's face in both the autism and control groups. This effect was significant in both the number of fixations and the total fixation time. Gaze to the mouth and other regions of the face did not vary significantly between the autism and control groups, and was not affected by oxytocin (Figure 3). Within the autism group, it was also found that oxytocin had a greater effect on looking time in individuals who exhibited lower eye contact in the placebo condition.

The findings in this study are supported by earlier work where oxytocin was shown to increase gaze to the eye region of static photographs in typical males^14^ and in autism.^29^ This latter study also found that oxytocin promotes social behavior in a computer-simulated ball game where the level of social interaction exhibited by the other players was varied.^29^ In the present study, we found that this benefit can be extended to a real-life, naturalistic discussion with another individual.

The group differences observed in gaze to the eye region also replicate findings reported in clinical assessments, where eye contact is substantially reduced in individuals with autism.^37^ Results within the autism group suggest that participants with the lowest levels of eye contact could benefit the most from oxytocin, with a greater increase in time spent looking at the eye region of the face (in line with a greater effect in autism versus controls). However, this analysis is necessarily limited by the size of the subgroups examined. It would be helpful to verify this finding in a study where repeated baseline (placebo) measures are possible in the autism group. It is also not known if these results would extend to individuals with greater impairment, as the autism group in this study only included individuals whose cognitive functioning was intact.

The current data support a wider body of work suggesting that neuropeptides have a role in everyday social behavior, and that the intranasal administration of oxytocin is able to effect measureable changes in brain and behavior. Interestingly, these effects of oxytocin in enhancing gaze towards the eyes in a naturalistic social interaction mimic the opposite effect previously observed in a patient with amygdala lesions.^38^ Separately, oxytocin was found to enhance reflexive gaze shifts towards the eyes, which in turn were associated with increased amygdala response.^21^ In combination with these findings, other evidence pointing towards a role of oxytocin in amygdala-mediated neural systems is growing.^10, 18, 20, 23, 24, 39^ It has also been suggested that oxytocin could reduce anxiety through amygdala-dependent mechanisms,^40, 41^ which may provide an additional benefit in social interactions.^33^

This study demonstrates that a single dose of oxytocin can have an effect on eye contact within a relatively short time window. It is not currently known whether this effect could be long lasting. However, it has also been suggested that oxytocin could promote longer lasting changes in social cognition such that when similar situations are encountered again they will be both more rewarding (through dopamine action) and more memorable (through noradrenaline action).^42^ Further research is needed in this area, though the concept may also be supported through animal models of oxytocin action, which continue to evolve.^42^

Other work is also needed to help understand how the effects observed in the present study might affect women, or translate into different environments in men. In particular, it would be useful to measure eye contact in other settings and with people who the individuals with autism are familiar with. Further research could also examine how oxytocin affects individuals who are more severely impaired, and to look at whether the results obtained in this study replicate in larger samples. The long-term effects of oxytocin and possible side effects from repeated doses should be examined.

In conclusion, this study demonstrates the potential for a single dose of 24 IU intranasal oxytocin to increase gaze to the eye region, an important marker for social communication difficulties that are characteristic of autism. The effect was significant within both the autism and control groups, with those in the autism group who spent less time looking to the eye region benefiting the most from oxytocin administration. The study also demonstrated the capability to quantitatively measure reduced eye contact in male adults with autism by using a novel measure of eye contact during a naturalistic social interaction. These results suggest that this technique is an ecologically valid and sensitive assay that demonstrates the influence of oxytocin on important social behaviors for autism. With further research, it is hoped that the current findings will assist the evaluation of oxytocin as a potentially therapeutic agent for enhancing aspects of social behavior in autism.

## Figures and Tables

**Table 1:** Participant characteristics

*Variable*	*Autism group (*n=*32)*			*Control group (*n=*34)*			*Group differences*	
	*Mean*	*s.d.*	*Range*	*Mean*	*s.d.*	*Range*	t	P*-value*
Age	36.04	9.36	18.50–56.00	32.80	9.35	21.17–52.92	1.447	0.153
Verbal IQ	110.44	15.13	75–147	110.82	17.63	67–140	0.030	0.976
Performance IQ	120.25	12.12	89–138	122.34	8.22	102–136	0.967	0.337
Full IQ	116.84	12.85	85–147	118.06	11.47	94–141	0.476	0.636

Abbreviation: IQ, intelligence quotient using the Wechsler Abbreviated Scale of Intelligence.
