# Supplementary material for: Oxytocin increases eye contact during a real-time, naturalistic social interaction in males with and without autism
Source: Transl Psychiatry. 2015 Feb 10;5(2):e507–. doi: 10.1038/tp.2014.146 (PMC4445747; doi:10.1038/tp.2014.146)
Supplement: Supplementary Tables [file tp2014146x1.doc]

**Supplemental Material**

### Supplementary Table 1. Differences between Autism and Controls in looking patterns (Placebo only)

|  | Number of Fixations (per second) | | | | | | | |
| --- | --- | --- | --- | --- | --- | --- | --- | --- |
|  | Autism Group (n=32) | | | Control Group (n=34) | | | Group Differences | |
| AOI | Mean | SD | Range | Mean | SD | Range | t-value | p-value |
| Eyes | 0.59 | 0.38 | 0.05-1.27 | 0.83 | 0.34 | 0.17-1.48 | 2.660 | 0.010 |
| Mouth | 0.47 | 0.32 | 0-1.44 | 0.48 | 0.25 | 0.08-0.98 | 0.243 | 0.809 |
| Other | 0.47 | 0.24 | 0.09-1.19 | 0.50 | 0.25 | 0.08-1.24 | 0.461 | 0.647 |
|  | Fixation Time (proportion) | | | | | | | |
|  | Autism Group (n=32) | | | Control Group (n=34) | | | Group Differences | |
| AOI | Mean | SD | Range | Mean | SD | Range | t-value | p-value |
| Eyes | 0.32 | 0.23 | 0.03-0.78 | 0.44 | 0.18 | 0.06-0.74 | 2.454 | 0.017 |
| Mouth | 0.26 | 0.21 | 0-.078 | 0.29 | 0.19 | 0.04-0.75 | 0.545 | 0.588 |
| Other | 0.22 | 0.13 | 0.03-0.61 | 0.26 | 0.15 | 0.03-0.68 | 1.160 | 0.250 |

### Supplementary Table 2. Reported Side Effects

|  | Autism (n=32) | | Controls (n=34) | |
| --- | --- | --- | --- | --- |
| Symptom | Oxytocin | Placebo | Oxytocin | Placebo |
| Runny Nose | 3 | 5 | 6 | 4 |
| Tiredness | 2 | 3 | 4 | 3 |
| Sore Throat | 1 | 0 | 0 | 0 |
| Shakiness | 0 | 1 | 0 | 0 |
| Mild Nausea | 0 | 0 | 0 | 1 |
